# Practical 1-Methylcyclopropene Technology for Increasing Apple (*Malus domestica* Borkh) Storability in the Aksu Region

**DOI:** 10.3390/foods13182918

**Published:** 2024-09-15

**Authors:** Shuang Zhang, Yuanqing Li, Meijun Du, Xihong Li, Junbo Wang, Zhaojun Ban, Yunhong Jiang

**Affiliations:** 1 Zhejiang Provincial Key Laboratory of Chemical and Biological Processing Technology of Farm Products, Zhejiang Provincial Collaborative Innovation Center of Agricultural Biological Resources Biochemical Manufacturing, School of Biological and Chemical Engineering, Zhejiang University of Science and Technology, Hangzhou 310023, China; 2 State Key Laboratory of Food Nutrition and Safety, College of Food Science and Engineering, Tianjin University of Science and Technology, Tianjin 300457, China; 3 Aksu Youneng Agricultural Technology Co., Ltd., Aksu 843001, China; 4 Hangzhou FoodSci. Agricultural Technology Co., Ltd., Hangzhou 310051, China; 5 Department of Applied Sciences, Faculty of Engineering and Environment, Northumbria University, Newcastle Upon Tyne NE1 8ST, UK

**Keywords:** postharvest apple, *Malus domestica* Borkh, 1-MCP, delayed application, storage quality, volatile aroma components

## Abstract

In recent years, Aksu apple has become popular with consumers because of its unique texture and taste. At present, maintaining quality during storage is the key problem with the apples in the Aksu region. 1-Methylcyclopropene (1-MCP) can delay fruit senescence, so is widely used in fruit preservation. However, many factors affect the preservation effect of 1-MCP. The effects of 1-MCP concentration (0 µL·L^−1^, 1 µL·L^−1^, 3 µL·L^−1^, 5 µL·L^−1^, and 8 µL·L^−1^) and postharvest application time (0, 1 and 2 d after harvest) on the quality of stored apple were studied. It was found that 1 µL·L^−1^ 1-MCP was more beneficial in improving the quality of stored apples, reduced the respiration intensity and decay rate, increased the fruit firmness and total soluble solid content, and reduced the relative content of ester volatile aroma components. In addition, 1-MCP treatment applied at different postharvest times also affected the sensory quality and flavor of apples. The effect of 1-MCP treatment immediately after harvest was better.

## 1. Introduction

As a kind of ‘Fuji’ apple, Aksu apple is famous for its unique water core, sweetness, crispness, and rich nutritional value, all because of its unique geographical environment and cultivation techniques [1,2,3,4]. In practical operation, in order to make the sugar content in the apples is sufficient and improve the fruit flavor quality, Aksu apples are generally picked after the frost’s descent (late October). At this time, the temperature difference between day and night is large in the Aksu region, the fruit growth period is fully extended, and the apples are picked at low temperature, so that the water content of the fruit is ideal. However, the water core easily dissipates after harvesting [5], so the storage of Aksu apples is more difficult. Apples are typically climacteric fruits, with increased respiration intensity and increased ethylene biosynthesis during ripening [6]. The increase in ethylene occurs during the last phase of fruit ripening, so, in apples, it usually occurs after harvest. During fruit storage, a large amount of ethylene is released, which accelerates the postripening and senescence processes, resulting in the rapid loss of nutrients and a rapid decline in quality.

The regulation of ethylene production is an effective way to improve the postharvest quality of climacteric fruits. A variety of methods have been shown to effectively reduce the ethylene production and respiration rate in postharvest fruits, among which 1-methylcyclopropene (1-MCP), as a more effective ethylene antagonist, has been widely used in postharvest treatment of fruits due to its advantages of simple use and good effect. By preventing ethylene from binding to its receptor and by inhibiting the expression of key enzyme genes of ethylene biosynthesis, it can prolong the postripening and aging processes of fruits and achieve good preservation effects [7,8]. In addition, 1-MCP treatment was shown to affect the volatile aroma components of apples [9]. However, the application of 1-MCP sometimes has some negative effects. Lu et al. found that the application of 1-MCP reduced the composition of the main volatile compounds that make up the apple aroma [10]. A decline in the quality of stored fruit leads to a decline in its commodity value. The effects of 1-MCP are affected by various factors such as fruit cultivar, maturity, and 1-MCP concentration [11,12,13]. The concentration of 1-MCP affects its effectiveness. Kwon et al. found that 0.5–1 µL·L^−1^ 1-MCP treatment improved the quality of apple fruit during cold storage, regardless of single or double treatment [14]. Previous studies also found that that application of 1-MCP at a concentration of 50–500 µL·L^−1^ could reduce chilling injury and peel pitting in citrus fruit, but 1-MCP concentrations higher than 1 mg·L^−1^ promoted decay through the development of rot in citrus fruits [11]. Moreover, if 1-MCP treatment cannot be carried out in time after harvest, and the delay also has a certain impact on the quality of stored fruit [15,16,17]. DeEll et al. indicated that minimizing 1-MCP treatment delay could reduce disorders in ‘McIntosh’ apples [18].

This study focused on the respiration intensity, firmness, decay rate, total soluble solid content, and aroma components of Aksu apples treated with 1-MCP at different concentrations and at different times after harvest. The effects of 1-MCP concentration and delayed treatment on storage quality of apple fruit were investigated in order to find out the best technical parameters and rules of its regulation on storage quality of apple fruit after harvest.

## 2. Materials and Methods

### 2.1. Fruit Materials and Treatments

All the tested apples (*Malus domestica* Borkh) were harvested on local farms (80°20′ E, 41°28′ N, Aksu Prefecture, Xinjiang) within a week after frost’s descent (25–30 October). Apples of uniform size as well as free of pests and mechanical damage were selected for the subsequent two experimental treatments.

The 500 apples were loaded into baskets after harvesting, and the fruits were fumigated immediately with 1-MCP in plastic sheds at different concentrations: 0 µL·L^−1^ (CT); 1 µL·L^−1^ (M1); 3 µL·L^−1^ (M3); 5 µL·L^−1^ (M5); 8 µL·L^−1^ (M8). After fumigation for 12 h, the samples were transported to the laboratory.

The other three groups of fruit samples: one group of fruits picked immediately (T0), one group of fruits that were placed at room temperature for 24 h after harvesting (T1), and one group of fruits that were placed at room temperature for 48 h after harvesting (T2), 100 fruits in each group, were fumigated with 1 µL·L^−1^ 1-MCP in plastic sheds for 12 h, then transported to the laboratory.

Immediately after arrival, we unpacked and precooled the apples at 0 °C for 24 h. They were then placed in a precise-temperature-controlled cold storage at 0 °C ± 0.1 °C, and the humidity was 90–92%. At the sampling time, each group of fruits was randomly sampled for the corresponding index determination.

### 2.2. Respiration Intensity

Respiration intensity was measured and calculated by referring to the methods of Du et al. [19], and a respiration intensity tester (GXH-3051H, JUN-FANG-LIHUA Technology-research Institute, Beijing, China) was used. Each group contained 3 fruits at each sampling time. The calculation formula is as follows:

X = ((W_2_ − W_1_) × V × M × 1000)/(V_0_ × m × t), (1)

where X is respiration intensity (mg CO_2_·kg^−1^·h^−1^); W_1_ is the CO_2_ content in the blank tank (%); W_2_ is the total CO_2_ content of the breathing tank after determination (%); V is the total volume of the breathing tank (L); M is the molar mass of CO_2_ (g·mol^−1^); V_0_ is the molar volume of CO_2_ at room temperature (L·mol^−1^); m is fruit weight (kg); t is the measurement time (h).

### 2.3. Firmness

Three apples were randomly selected from each group, and the apples were cut along the equatorial part. The fruit firmness was measured three times using a digital force gauge (GY-4, Top Yunnong Technology Co., Ltd., Hangzhou, China) with an 11 mm probe perpendicular to the cross-section. The average value was taken as the firmness value of the treated fruit, and the unit was kg·cm^−2^.

### 2.4. Decay Rate

Fruit decay is expressed as the percentage of fruit with visible fungal development. Ten fruits from each treatment were used for analysis. The decay rate of the apples was calculated by the counting method.

Decay rate = (The number of decayed fruit)/(The total number of fruit) × 100%(2)

### 2.5. Total Soluble Solid (TSS) Content

Three apples were selected from the equatorial position, cut into pieces, and then juiced. The TSS content of the apples was determined with a Pocket Brix-Acidity Meter (PAL-3, Atago Co., Ltd., Tokyo, Japan).

### 2.6. Volatile Component Analysis

According to Wang et al. [20], 40 g of the pulp was weighed, and the volatile aroma components of the fruit were collected by headspace solid-phase microextraction and determined by gas chromatography–mass spectrometry (GCMS-QP2010 Ultral, Shimadzu, Tokyo, Japan). The carrier gas was pure helium with a flow rate of 1.0 mL min^−1^. The programmed temperature was 40 °C for 2 min; then increased to 150 °C at 4 °C min^−1^ and maintained for 2 min; increased at 8 °C min^−1^ to 250 °C and maintained for 6 min. The temperature of the quadrupole mass spectrometry detector was 150 °C, the ion source was 280 °C, and the transfer line was 250 °C. Finally, the relative mass percentages of the compounds were quantitatively determined by peak area normalization.

### 2.7. Statistical Analysis

All experiments were performed with three replications, and the data were statistically analyzed using SPSS 24.0 and plotted using Origin 9.0. All data are expressed as mean ± standard deviation (SD), and one-way analysis of variance (ANOVA) was performed. Duncan’s test was used to compare the mean values, and *p* < 0.05 was considered a significant difference.

## 3. Results

### 3.1. Different Concentrations of 1-MCP Affected the Quality of Stored Apple

#### 3.1.1. Effects of Different Concentrations of 1-MCP on Quality of Stored Apple

**Figure 1:**
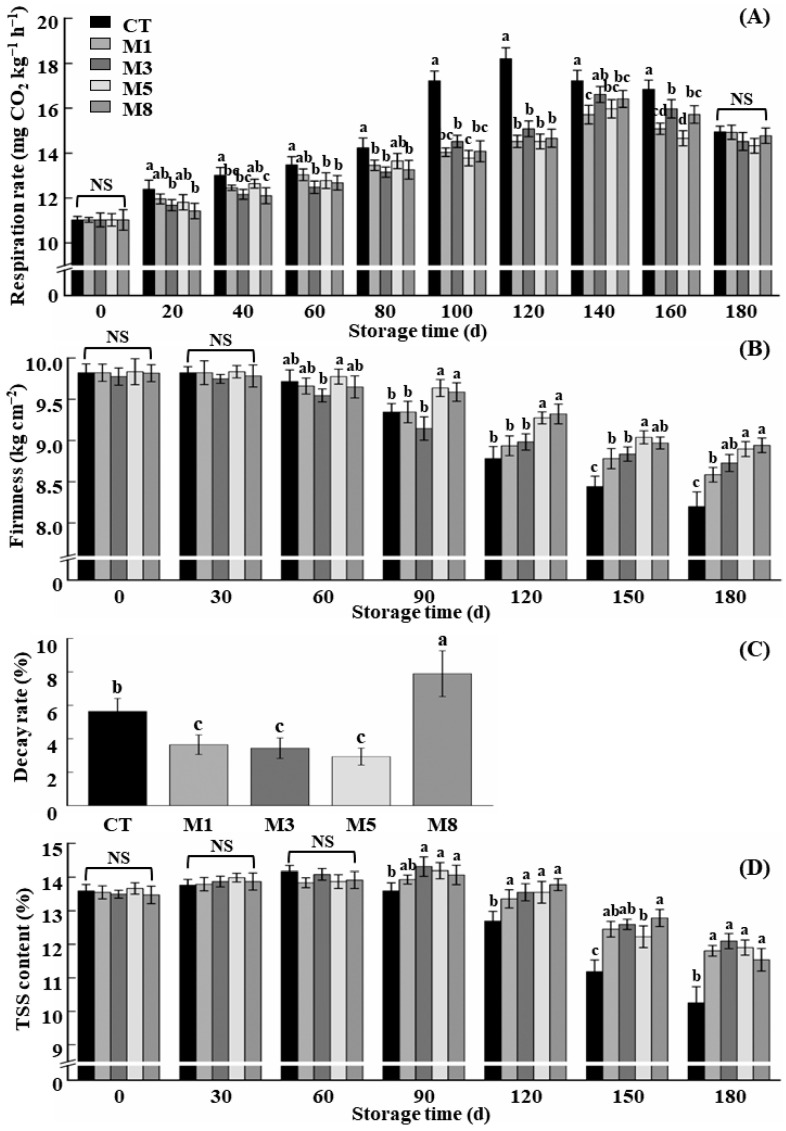
Effects of different concentrations of 1-MCP on the quality of stored apple. (**A**) Respiration intensity; (**B**) firmness; (**C**) decay rate; (**D**) total soluble solid (TSS) content. CT, M1, M3, M5, and M8 were treated with 1-MCP concentrations of 0 µL·L^−1^, 1 µL·L^−1^, 3 µL·L^−1^, 5 µL·L^−1^, and 8 µL·L^−1^, respectively. The vertical bars indicate ± standard deviation of the means (*n* = 3). Different letters indicate significant differences among different treatments at *p* < 0.05. NS means no significant difference.

As can be seen in Figure 1A, the respiration intensity of apples treated with 1-MCP was decreased significantly during storage compared with that of the CT group. The peak of respiration of fruits in the CT group appeared at 120 d, while the peak respiratory time of the fruits in the other four groups treated with 1-MCP was 140 d, indicating a delay.

The firmness of the fruit is one of the basic attributes that can best reflect the commodity value of apples. As can be seen from Figure 1B, the firmness of the apples decreased continuously during storage, and 1-MCP effectively delayed the decline in fruit firmness compared with that of the CT group. The higher the concentration of 1-MCP, the better the fruit firmness retention effect. After 180 days of storage, the fruit firmness in the 1-MCP treatment group was 4.76% (M1), 5.73% (M3), 8.55% (M5), and 9.03% (M8) higher than that in the CT group, respectively.

Figure 1C shows the effect of different concentrations of 1-MCP on the fruit decay rate of the apples during 180 days of storage. Fruit decay rates in the groups treated with 1 µL·L^−1^, 3 µL·L^−1^ and 5 µL·L^−1^ 1-MCP concentrations were significantly reduced by 2.0%, 2.2%, and 2.7% compared with those of the CT group, respectively. However, when the concentration of 1-MCP was 8 µL·L^−1^, the fruit decay rate was increased by 2.3% compared with that of the CT group. Too high a 1-MCP concentration was not conducive to the inhibition of fruit decay.

As can be seen from Figure 1D, the TSS content of the apples basically showed a trend of slowly increasing at first and then rapidly decreasing. Compared with that of the CT group, 1-MCP significantly delayed the process of fruit ripening and aging, and the peak TSS content of the fruits treated with 1-MCP was delayed compared with that of CT group. After 180 days of storage, the TSS contents of fruits in 1-MCP treatment groups (M1, M3, M5, and M8) were 1.5%, 1.8%, 1.6% and 1.3% higher than those of the CT group, respectively. However, there was no significant difference in TSS maintenance effect of different concentrations of 1-MCP (*p* > 0.05).

#### 3.1.2. Effects of Different Concentrations of 1-MCP on Volatile Aroma Components of Apple

**Figure 2:**
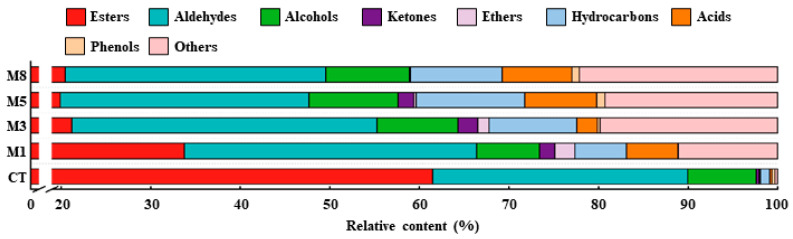
Effects of different concentrations of 1-MCP on volatile aroma components of apple after 180 d of storage. CT, M1, M3, M5, and M8 were treated with 1-MCP concentrations of 0 µL·L^−1^, 1 µL·L^−1^, 3 µL·L^−1^, 5 µL·L^−1^, and 8 µL·L^−1^, respectively.

Figure 2 shows the differences in the volatile components of the apples treated with different concentrations of 1-MCP and stored for 180 days. It can be seen from Table 1 and Figure 2 that when the apples was stored for 180 days, the volatile components of the fruit (CT group) were esters, alcohols and aldehydes, accounting for more than 97% of the total volatile components, and their relative contents were 61.5%, 7.62%, and 28.46%, respectively. As shown in Table 1, 1-MCP treatment significantly inhibited the ester components in the fruits, which may have been related to the subsequent physiological chain reaction blocked by 1-MCP inhibiting ethylene synthesis and binding to its receptor protein. The 1 µL·L^−1^ 1-MCP treatment significantly reduced the ester components of the fruits by 45.09% (Table 1), and the higher concentration of 1-MCP showed a greater inhibitory effect. In addition, the aldehydes in the fruits in the group treated with 1–3 µL·L^−1^ 1-MCP were slightly higher than those in the CT group (Table 1), while the higher 1-MCP concentration had little effect on aldehydes. For alcohols, 1-MCP at a 1 µL·L^−1^ concentration had little effect on the alcohol components (Table 1), while the proportion of alcohol components increased slightly at higher 1-MCP concentrations (the M3, M5, and M8 groups).

### 3.2. Different Times of 1-MCP Treatment Affected the Quality of Stored Apple

#### 3.2.1. Effects of Different Treatment Times with 1-MCP on Quality of Stored Apple

**Figure 3:**
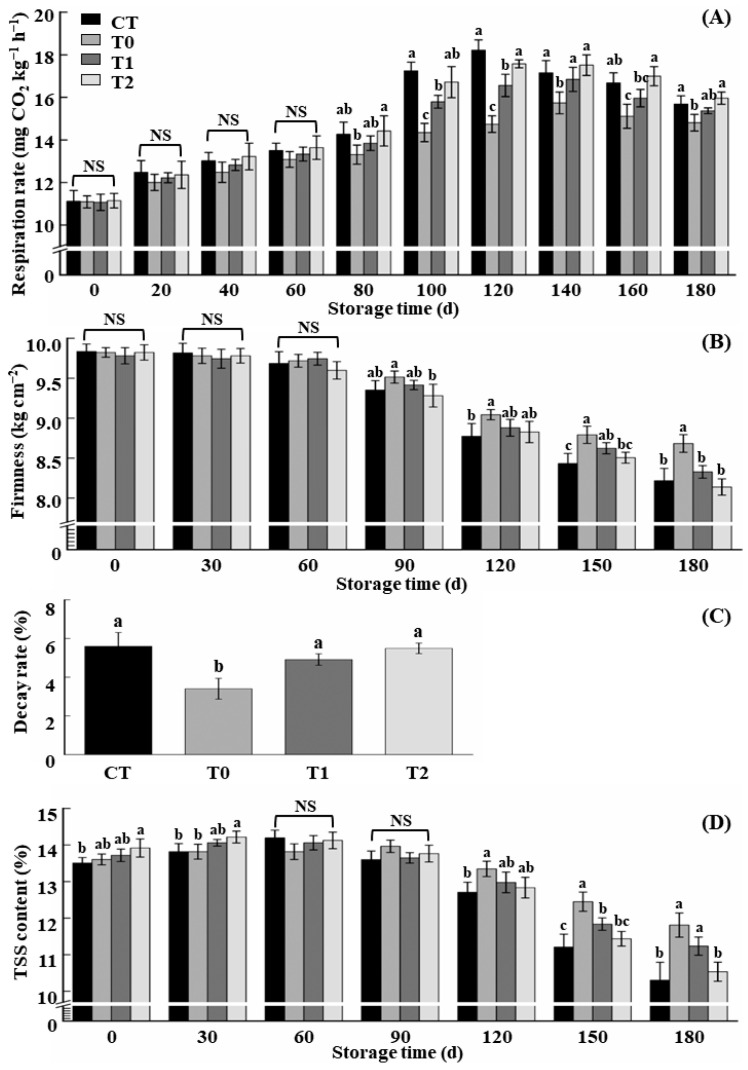
Effects of different treatment times with 1-MCP on the quality of stored apple. (**A**) Respiration intensity; (**B**) firmness; (**C**) decay rate; (**D**) total soluble solid (TSS) content. CT was treated without 1-MCP; T0, T1, and T2 were treatments with 1-MCP delayed for 0, 1, and 2 days, respectively. The vertical bars indicate ± standard deviation of the means (*n* = 3). Different letters indicate significant differences among different treatments at *p* < 0.05. NS means no significant difference.

As can be seen in Figure 3A, the immediate postharvest treatment inhibited the overall respiratory intensity and effectively delayed the appearance of the respiratory peak. Although the fruits were treated after 24 h, the treatment also showed a certain effect on inhibiting respiration and reducing the peak of respiration, but the inhibitory effect was weaker than that of immediate 1-MCP treatment (the T0 group); however, there was no significant difference between the CT group and the T2 group.

As can be seen in Figure 3B, the earlier the treatment time with 1-MCP, the slower the decline in fruit firmness, and the immediate treatment with 1-MCP after harvest (the T0 group) had the best effect on delaying the decline in fruit firmness, and the firmness of the fruits in this group was significantly increased by 5.41% compared with that in the CT group after 180 days of storage. However, compared with the CT group, 1-MCP treatment after 24 h and 48 h at room temperature showed no significant effect on the maintenance of fruit firmness (*p* > 0.05).

As can be seen in Figure 3C, the decay rate of the apples fumigated with 1 µL·L^−1^ 1-MCP immediately after harvest was the lowest at 180 days of storage, only 3.4%, which was significantly lower than that of the CT group by 2.2% (*p* < 0.05). The decay rate of fruits treated with 1-MCP after 24 h and 48 h stored for 180 days was 4.9% and 5.5%, which were 0.7% and 0.1% lower than that of the CT group, respectively, but were not significantly different (*p* > 0.05).

As can be seen in Figure 3D, the maintenance effect of 1-MCP on TSS negatively correlated with the treatment time: when this treatment time was delayed to 48 h, the maintenance of fruit TSS was almost ineffective. Compared with that of the CT group, the TSS level of the fruits in the T0 and T1 groups was always higher, and, after 180 days of storage, the TSS level of the fruits in the T0 and T1 groups was effectively increased by 1.6% and 1.1%, respectively, and the effect of the former was significant (*p* < 0.05). However, the TSS of the fruits in the T2 group was only 0.2% higher than that in the CT group, indicating the treatment hardly improved the quality of the stored fruits.

#### 3.2.2. Effects of Different Delayed 1-MCP Treatment of on Volatile Aroma Components of Apple

Table 2 showed the effect of 1-MCP at a 1 µL·L^−1^ concentration applied at different times on the composition of the volatile substances in the apples stored at phase temperature for 180 days. Obviously, delayed postharvest 1-MCP treatment had a great effect on the esters of the apples. After delaying treatment for 48 h, the ester substances in the fruits were reduced by 2.1% compared with those of the CT group. Compared with the immediate postharvest 1-MCP treatment (T0 group), delayed postharvest treatment increased the relative content of the alcohols in the apples, which was consistent with the CT treatment. On the contrary, delaying postharvest treatment (the T1 and T2 groups) reduced the relative contents of aldehydes, ketones, ethers, hydrocarbons, and acids.

## 4. Discussion

### 4.1. Different Concentrations of 1-MCP Affected the Quality of Stored Apple

1-MCP inhibits physiological activities such as postharvest respiration in fruit [21,22]. 1-MCP treatment reduced the respiration intensity of apple during storage. Also a climacteric fruits, Mata et al. took tomato as the research object and found that 1-MCP treatment reduced the expression level of alternative oxidase gene, resulting in reduced cellular respiration and an inhibited climacteric rise in CO_2_ production [23]. And, there was no significant difference in the inhibitory effect of 1-MCP among different concentrations, which may have been due to the fact that the binding degree of 1-MCP with the competitive receptor protein of fruit ethylene reached saturation when the concentration of 1-MCP was 1 µL·L^−1^. Although the inhibitory effect was slightly stronger when the concentration was higher, this effect was minimal and had little effect on the respiration of the fruit. Therefore, 1-MCP at a 1 µL·L^−1^ concentration significantly inhibited postharvest respiration. The postharvest softening of fruit is a normal physiological phenomenon in during postharvest storage, which is the result of cell senescence, cell wall degradation, and other factors [13]. Compared with the CT group, 1-MCP increased the firmness of the apples and inhibited the softening of the fruit. Win et al. also reached the same conclusion in two apple cultivars, showing that 1-MCP maintained cell wall pectin and delayed softening by reducing the solubilization of polyuronides and neutral sugars and limiting the activities of cell wall hydrolysis [13]. Rahman et al. reported that apples treated with 1 µL·L^−1^ 1-MCP had higher firmness during storage than apples treated with 0.5 µL·L^−1^ 1-MCP, and the higher firmness may have been due to the lower catabolism in apples and the reduced degradation of complex carbohydrates [24]. When the concentration of 1-MCP was higher than 5 µL·L^−1^, there was no significant difference in fruit firmness, indicating that 1-MCP also has a certain threshold range for the maintenance of fruit firmness. When the concentration of 1-MCP was 1–5 µL·L^−1^, there was a positive correlation on the retention of firmness of apples.

Within a certain concentration range, 1-MCP could effectively reduce fruit decay and control ethylene-stimulated rots [11]. However, 1-MCP concentrations higher than 1 mg·L^−1^ promote the development of rot decay in citrus fruits [11]. This is similar to our findings on the decay rate of apples. Total soluble solid (TSS) is one of the basic indices that can best represent the nutritional quality of apples, and TSS content is related to apple flavor. The TSSs in apples are affected by many factors, the influences of which are inconclusive [25]. Regarding the change in the TSS content in apples during storage, the postripening of fruit in the early stage is the dominant factor, while the aging and degeneration processes are the main factors in the later stage. The increase in TSS is due to the conversion of complex polysaccharides and starches into simple sugars during ripening and senescence [26]. Rahman et al. pointed out that 1-MCP treatment delayed fruit senescence, thereby maintaining TSS [24]. The 1 µL·L^−1^ concentration of 1-MCP could maintain the TSS content at a high level during storage.

Fruit aroma depends on the type and concentration of volatile components that affect sensory properties [27], and, during fruit ripening, a blend of aromatic compounds is produced [9]. Aroma is one of the important flavor properties of apples, and different kinds of apples have different characteristic volatile components. The flavorful substances in apples are mainly esters, aldehydes, and alcohols. A large number of studies have shown that 1-MCP treatment affects the aroma of fruits such as kiwifruit [27], apple [28], and peach [29]. The characteristic volatile components of apple are 2-methyl-butyl acetate, n-butyl acetate, hexyl acetate, n-hexyl alcohol, 2-methyl-1-butanol, and trans-2-hexenal. The effects of 1-MCP on fruit esters are greater, followed by alcohols and aldehydes. This may be related to fatty acid metabolism [30]. Previous studies showed that only specific steps of the volatile component pathway are controlled by ethylene, and, in ethylene-suppressed apples, only esters and alcohols are effectively reduced [9]. 1-MCP treatment inhibited the production of esters, and Wang et al. also found a similar phenomenon in postharvest kiwifruit, which could be attributed to its inhibition of the expression levels of the genes involved in the lipoxygenase pathway [27]. The application of 1-MCP can inhibit the production of ester volatile components and make the fruit aroma weaker. 1-MCP at 1–3 µL·L^−1^ concentrations can maintain the aldehyde aroma components to a certain extent, and the inhibition and maintenance of aroma at higher concentrations are somewhat ineffective, so there is a risk of stimulating the production of adverse gases.

### 4.2. Different Delayed 1-MCP Treatment Times Affected the Quality of Stored Apple

Compared with preharvest or preharvest and postharvest combined treatment, postharvest 1-MCP treatment was sufficient in maintaining the quality of stored apples [26]. Delaying the treatment time with 1-MCP after harvest also had a certain effect on the physiological state of the apples during storage. The earlier the 1-MCP treatment of the fruits, the better the inhibition effect on respiratory physiological activities during storage, and the immediate postharvest treatment had the best inhibition effect. This result is similar to that of Nguyen et al. [31], who found that fruit ethylene and respiratory CO_2_ production increased with postharvest application time delay. This may be due to delayed postharvest treatment, which increases the chances of ethylene and receptor binding [16]. Meanwhile, 1-MCP treatment can effectively delay the decline in apple firmness during postharvest storage, but there are certain differences in the maintenance effect of 1-MCP treatment on the firmness of apples at different times. Through our research, we found that 1-MCP fumigation should be used immediately after harvest to delay the firmness decline during storage, and delaying the postharvest treatment time reduces the effect of 1-MCP. This conclusion is related to many factors such as fruit varieties. Nguyen et al. pointed out that1-MCP treatment of pear fruits could be delayed until 3 d after harvest, and delaying treatment to 5 and 7 d after harvest was too late to control postharvest ripening [16].

The immediate treatment of fruits with 1-MCP after harvest could inhibit physiological diseases and decay by regulating physiological metabolism levels, which had a significant positive effect on inhibiting fruit rot and deterioration. The earlier the 1-MCP treatment time, the better the TSS maintenance. This result is also consistent with the results of the above studies: delaying the treatment of fruits with 1-MCP for 48 h cannot effectively improve the TSS content or maintain the taste quality of apples during storage. Lu et al. indicated that delaying the 1-MCP treatment of apples resulted in poor scald control [32]. In addition, delayed postharvest 1-MCP treatment affected the volatile aroma components of the apples. In short, in order to improve the storage quality and extend the storage period of apples, it is best to treat them immediately after harvest.

## 5. Conclusions

Treatment with 1-MCP and delayed postharvest treatment time improved the quality of apples to varying degrees. The appropriate concentration of 1-MCP reduced the postharvest respiration intensity and decay of the apples and improved the fruit firmness and TSS quality, but it produced negative effects, inhibiting the production of volatile aroma components, especially esters. When the 1-MCP concentration exceeded 3 μL·L^−1^, it negatively affected the fruit and increased the risk of metabolic disorders and decay. It is better to use 1 µL·L^−1^ 1-MCP to treat the apples. 1-MCP treatment 48 h after harvest was not conducive to improving the quality of the stored apples. Therefore, in order to improve the quality of stored Aksu apple fruit, it is best to treat apples with 1-MCP immediately after harvest.

## Figures and Tables

**Table 1:** Effects of different concentrations of 1-MCP on esters, aldehydes, and alcohols in apple after 180 d of storage.

No.	Aroma Chemical Name	Relative Content/%				
		CT	M1	M3	M5	M8
**Esters**						
1	Propyl acetate	0.09	0.03	0.02	0.02	—
2	Isobutyl acetate	0.3	0.4	0.09	—	—
3	N-butyl acetate	9.76	3.27	4.23	2.74	3.54
4	2-Methylbutyl acetate	24.39	9.24	5.27	6.24	7.75
5	Propyl butyrate	0.72	0.02	—	—	—
6	Butyl propionate	0.62	1.23	1.22	1.54	0.09
7	Propyl propionate	0.2	0.57	0.27	—	—
8	Amyl acetate	2.3	1.5	0.3	0.1	0.3
9	Prenyl acetate	0.08	—	—	—	—
10	Propyl 2-methyl butyrate	0.26	0.34	0.17	0.21	0.03
11	Isobutyl butyrate	0.18	0.02	0.01	0.02	0.04
12	2-Pentanol propanoate	0.14	0.14	0.15	0.14	0.16
13	Isobutyl butyrate	1.28	0.72	1.56	0.22	0.04
14	Ethyl hexanoate	0.23	—	—	0.02	0.03
15	Butyl acetate	1.77	0.12	0.34	0.02	—
16	Hexyl acetate	12.29	8.72	6.13	7.43	6.71
17	Ethyl 2-methylbutanoate	0.18	0.01	0.02	0.07	—
18	N-butyl 2-methylbutyrate	1	—	—	—	—
19	Isoamyl butyrate	0.07	—	—	0.12	0.23
20	2-Methylbutyl butyrate	0.15	0.32	0.02	—	—
21	Hexyl butyrate	0.35	0.32	—	—	—
22	Butyl butyrate	2.85	3.75	0.05	0.07	1.24
23	Hexyl 2-methylbutyrate	0.24	—	—	—	—
24	Diisobutyl phthalate	0.15	—	1.22	0.85	0.22
25	Dibutyl phthalate	0.52	1.25	0.07	0.02	0.02
26	Di(2-ethylhexyl) adipate	1.12	1.76	0.05	0.07	0.04
27	Bis(6-methylheptyl) phthalate	0.26	0.04	0.02	—	—
All		61.5	33.77	21.21	19.9	20.44
**Aldehydes**						
1	3-Hexenal	1.6	2.73	3.21	1.79	2.22
2	Hexanal	4.18	4.72	3.12	2.98	3.03
3	Phenylacetaldehyde	—	0.09	0.13	0.22	0.14
4	Trans-2-hexenal	14.08	17.54	13.21	12.22	9.97
5	N-caprylic aldehyde	0.13	—	—	0.12	0.07
6	Nonanal	0.3	0.12	0.2	—	—
7	Decanal	0.25	0.24	0.27	0.22	0.31
8	5-Hydroxymethylfurfural	4.29	2.72	0.21	0.19	1.12
9	Acetaldehyde	2.27	4.35	6.72	4.57	5.52
10	2-Hexenal	1.24	0.07	4.77	3.57	6.72
11	2-Bromooctadecanal	0.12	0.02	2.23	1.89	0.02
All		28.46	32.6	34.07	27.77	29.12
**Alcohols**						
1	1-Butanol	0.06	—	—	0.88	1.72
2	1-Hexanol	2.72	3.57	1.66	0.02	0.05
3	2-Methyl-1-butanol	2.54	1.78	2.01	1.95	1.23
4	2-Ethyl-1-hexanol	1.28	0.45	1.23	1.53	1.22
5	Isooctyl alcohol	0.87	0.02	0.02	1.27	0.45
6	Linalool	0.15	0.03	0.01	1.12	0.02
7	1-Hexanol	—	1.12	0.02	0.07	0.04
8	1,3-Octanediol	—	—	0.09	0.09	0.02
9	8-Heptadecanol	—	0.03	—	0.14	1.11
10	1-Methyl-1-pentanol	—	0.05	1.75	1.02	2.24
11	Trans-2-hexene-1-ol	—	—	2.22	1.87	1.22
All		7.62	7.05	9.01	9.96	9.32

“—” indicates that the volatile component was not detected. CT, M1, M3, M5, and M8 were treated with 1-MCP concentrations of 0 µL·L^−1^, 1 µL·L^−1^, 3 µL·L^−1^, 5 µL·L^−1^, and 8 µL·L^−1^, respectively. Relative content refers to the relative mass percentages of volatile aroma components quantitatively determined by peak area normalization.

**Table 2:** Effects of different delayed 1-MCP treatment time on volatile components of apple after 180 d of storage.

		CT	T0	T1	T2
**Esters**	Numbers	31	20	24	27
	Relative content/%	61.5	33.77	52.3	59.4
**Alcohols**	Numbers	6	8	5	5
	Relative content/%	7.62	7.05	8.02	7.53
**Aldehydes**	Numbers	10	10	13	11
	Relative content/%	28.46	32.6	30.3	27.1
**Ketones**	Numbers	2	2	1	1
	Relative content/%	0.36	1.72	0.12	0.05
**Ethers**	Numbers	1	3	0	0
	Relative content/%	0.13	2.23	0	0
**Hydrocarbons**	Numbers	4	4	5	2
	Relative content/%	1.01	5.75	1.12	0.78
**Acids**	Numbers	2	5	3	3
	Relative content/%	0.29	5.74	2.32	0.55
**Phenols**	Numbers	1	1	0	1
	Relative content/%	0.32	0.02	0	0.03
**Others**	Numbers	6	4	5	5
	Relative content/%	0.31	11.12	5.82	4.56

CT was not treated with 1-MCP, and T0, T1, and T2 received 1-MCP treatments that were delayed by 0, 1, and 2 days, respectively.

## Data Availability

The original contributions presented in the study are included in the article, further inquiries can be directed to the corresponding author.

## References

[1] Du M., Liu Z., Zhang X., Li H., Liu Z., Li X., Song J., Jia X., Wang L. (2021). Effect of Pulsed Controlled Atmosphere with CO_2_ on the Quality of Watercored Apple during Storage. Sci. Hortic..

[2] Li X., Liu Z., Ran Y., Li L., Chen L., Lin Q., Liang F., Li J., Li X., Tang Y. (2022). Short-Term High Oxygen Pre-Stimulation Inhibits Browning of Fresh-Cut Watercored Fuji Apples. Postharvest Biol. Technol..

[3] Sugiura T., Ogawa H., Fukuda N., Moriguchi T. (2013). Changes in the Taste and Textural Attributes of Apples in Response to Climate Change. Sci. Rep..

[4] Yang M., Lin Q., Luo Z., Ban Z., Li X., Reiter R.J., Zhang S., Wang L., Liang Z., Qi M. (2023). Ongoings in the Apple Watercore: First Evidence from Proteomic and Metabolomic Analysis. Food Chem..

[5] Algul B.E., Shoffe Y.A., Park D., Miller W.B., Watkins C.B. (2021). Preharvest 1-Methylcyclopropene Treatment Enhances ‘Stress-Associated Watercore’ Dissipation in ‘Jonagold’ Apples. Postharvest Biol. Technol..

[6] Zenoni S., Savoi S., Busatto N., Tornielli G.B., Costa F. (2023). Molecular Regulation of Apple and Grape Ripening: Exploring Common and Distinct Transcriptional Aspects of Representative Climacteric and Non-Climacteric Fruits. J. Exp. Bot..

[7] Costa F., Alba R., Schouten H., Soglio V., Gianfranceschi L., Serra S., Musacchi S., Sansavini S., Costa G., Fei Z. (2010). Use of Homologous and Heterologous Gene Expression Profiling Tools to Characterize Transcription Dynamics during Apple Fruit Maturation and Ripening. BMC Plant Biol..

[8] Watkins C.B. (2006). The Use of 1-Methylcyclopropene (1-MCP) on Fruits and Vegetables. Biotechnol. Adv..

[9] Tadiello A., Longhi S., Moretto M., Ferrarini A., Tononi P., Farneti B., Busatto N., Vrhovsek U., Molin A.D., Avanzato C. (2016). Interference with Ethylene Perception at Receptor Level Sheds Light on Auxin and Transcriptional Circuits Associated with the Climacteric Ripening of Apple Fruit (*Malus x Domestica* Borkh.). Plant J..

[10] Lu X., Meng G., Jin W., Gao H. (2018). Effects of 1-MCP in Combination with Ca Application on Aroma Volatiles Production and Softening of ‘Fuji’ Apple Fruit. Sci. Hortic..

[11] Dou H., Jones S., Ritenour M. (2005). Influence of 1-MCP Application and Concentration on Post-Harvest Peel Disorders and Incidence of Decay in Citrus Fruit. J. Hortic. Sci. Biotechnol..

[12] Satekge T.K., Magwaza L.S. (2022). Postharvest Application of 1-Methylcyclopropene (1-MCP) on Climacteric Fruits: Factors Affecting Efficacy. Int. J. Fruit Sci..

[13] Win N.M., Yoo J., Naing A.H., Kwon J.-G., Kang I.-K. (2021). 1-Methylcyclopropene (1-MCP) Treatment Delays Modification of Cell Wall Pectin and Fruit Softening in “Hwangok” and “Picnic” Apples during Cold Storage. Postharvest Biol. Technol..

[14] Kwon J.-G., Yoo J., Win N.M., Maung T.-T., Naing A.H., Kang I.-K. (2021). Fruit Quality Attributes of ‘Arisoo’ and ‘Picnic’ Apples as Influenced by 1-Methylcyclopropene Concentration and Its Application Frequency during Cold Storage. Horticulturae.

[15] Jung S.-K., Watkins C.B. (2008). Superficial Scald Control after Delayed Treatment of Apple Fruit with Diphenylamine (DPA) and 1-Methylcyclopropene (1-MCP). Postharvest Biol. Technol..

[16] Nguyen L.L.P., Pham T.T., Syium Z.H., Zsom-Muha V., Baranyai L., Zsom T., Hitka G. (2022). Delay of 1-MCP Treatment on Post-Harvest Quality of ‘Bosc Kobak’ Pear. Horticulturae.

[17] Satekge T.K., Magwaza L.S. (2022). Delayed 1-Methylcyclopropene Application Improves Ripening Recovery in Banana Fruit after Cold Storage. Hortic. Environ. Biotechnol..

[18] DeEll J.R., Ayres J.T., Murr D.P. (2008). 1-Methylcyclopropene Concentration and Timing of Postharvest Application Alters the Ripening of ‘McIntosh’ Apples during Storage. HortTechnology.

[19] Du M., Jia X., Li J., Li X., Jiang J., Li H., Zheng Y., Liu Z., Zhang X., Fan J. (2020). Regulation Effects of 1-MCP Combined with Flow Microcirculation of Sterilizing Medium on Peach Shelf Quality. Sci. Hortic..

[20] Wang H., Wang C., Cheng L., Chang Y., He P., Li L. (2017). Effect of Metaxenia on Volatile Compounds in Bagged Apple Fruit of Fuji. Agric. Sci. Technol..

[21] Jeong J., Huber D.J., Sargent S.A. (2002). Influence of 1-Methylcyclopropene (1-MCP) on Ripening and Cell-Wall Matrix Polysaccharides of Avocado (*Persea Americana*) Fruit. Postharvest Biol. Technol..

[22] Saquet A.A., Almeida D.P.F. (2017). Ripening Physiology and Biochemistry of ‘Rocha’ Pear as Affected by Ethylene Inhibition. Postharvest Biol. Technol..

[23] Mata C.I., Magpantay J., Hertog M.L.A.T.M., Van de Poel B., Nicolaï B.M. (2021). Expression and Protein Levels of Ethylene Receptors, CTRs and EIN2 during Tomato Fruit Ripening as Affected by 1-MCP. Postharvest Biol. Technol..

[24] Rahman W.U., Hashmi M.S., Durrani Y., Shah S., Ahmad A., Alam S., Ali W. (2022). Hypobaric Treatment Augments the Efficacy of 1-MCP in Apple Fruit. J. Food Sci. Technol..

[25] Tomala K., Małachowska M., Guzek D., Głąbska D., Gutkowska K. (2020). The Effects of 1-Methylcyclopropene Treatment on the Fruit Quality of ‘Idared’ Apples during Storage and Transportation. Agriculture.

[26] Tomala K., Guzek D., Głąbska D., Małachowska M., Widłak Ł., Krupa T., Gutkowska K. (2022). Maintaining the Quality of ‘Red Jonaprince’ Apples during Storage by 1-Methylcyclopropene Preharvest and Postharvest Treatment. Agriculture.

[27] Wang Q., An X., Xiang M., Chen X., Luo Z., Fu Y., Chen M., Chen J. (2021). Effects of 1-MCP on the Physiological Attributes, Volatile Components and Ester-Biosynthesis-Related Gene Expression during Storage of ‘Jinyan’ Kiwifruit. Horticulturae.

[28] Defilippi B.G., Dandekar A.M., Kader A.A. (2004). Impact of Suppression of Ethylene Action or Biosynthesis on Flavor Metabolites in Apple (*Malus Domestica* Borkh) Fruits. J. Agric. Food Chem..

[29] Tang J., Zhang W., Liu R., Liao Q., Jiang P., Li Z., Lan J. (2020). Effect of 1-Methylcyclopropene on the Aroma Volatiles, Polyphenols Contents, and Antioxidant Activity of Post-Harvest Ripening Peach (*Prunus persica* L.) Fruit. Qual. Assur. Saf. Crops Foods.

[30] Lv Y., Chen G., Ouyang H., Sang Y., Jiang Y., Cheng S. (2021). Effects of 1-MCP Treatment on Volatile Compounds and Quality in Xiaobai Apricot during Storage at Low Temperature. J. Food Process. Preserv..

[31] Nguyen L.P.L., Hitka G., Zsom T., Kókai Z. (2016). Application of 1-MCP on Apricots at Different Temperatures and Days after Harvest. Acta Aliment..

[32] Lu X., Nock J.F., Ma Y., Liu X., Watkins C.B. (2013). Effects of Repeated 1-Methylcyclopropene (1-MCP) Treatments on Ripening and Superficial Scald of ‘Cortland’ and ‘Delicious’ Apples. Postharvest Biol. Technol..

